# Subcutaneous golimumab induced and maintained clinical response in a child with a biological-experienced steroid-refractory flare of ulcerative colitis: A case report

**DOI:** 10.1097/MD.0000000000027283

**Published:** 2021-09-24

**Authors:** Marouf Alhalabi, Kamal Alaa Eddin, Khaled Cheha, Ahmad Abbas

**Affiliations:** Gastroenterology Department, Damascus Hospital, Damascus, Syria.

**Keywords:** anti-TNFα, case-report, children, golimumab, refractoriness, ulcerative colitis

## Abstract

**Introduction:**

Golimumab is a fully human antitumor necrosis monoclonal antibody that can be administered by either subcutaneous injection or intravenous infusion. Golimumab is approved for the treatment of the adults with rheumatic diseases, and ulcerative colitis, Whereas in children, golimumab is indicated only for the treatment of active polyarticular juvenile idiopathic arthritis. We have written on the off-label use of subcutaneous golimumab, which helped to induce and maintain remission on a low-weight biologically experienced child with steroid-refractory ulcerative colitis flare.

**Patient concerns:**

A 13-year-old pancolitis Syrian boy presented with abdominal pain and six to seven times bloody diarrhea. The child had treated with mesalamine 80 mg/kg/day, azathioprine 2.5 mg/kg/day, infliximab with an induction dose of 5 mg/kg at weeks 0, 2, and 6 followed by 5 mg/kg every 8 weeks. Infliximab did not maintain remission as the patient suffered from two flares that required hospital admission, intravenous corticosteroids, and infliximab escalation. Initial tests disclosed leukocytosis, anemia, hypoalbuminemia, an elevation in C-reactive protein and fecal calprotectin. All Stool studies were negative including routine stool cultures, *Clostridium difficile* toxin, *Escherichia coli* O157:H7, Cryptosporidium, and microscopy for ova and parasites. A sigmoidoscopy revealed multiple large ulcerations and spontaneous bleeding, colon biopsies were negative for *Clostridium difficile* and Cytomegalovirus. Cyclosporine, tacrolimus, and adalimumab were unavailable in Syria. Child's parents opposed colectomy as a treatment option.

**Diagnosis:**

Ulcerative colitis flare.

**Interventions:**

A subcutaneous golimumab with a loading dose of 200 mg at week 0, followed by 100 mg at week 2, then 50 mg every 4 weeks.

**Outcomes:**

The patient achieved clinical remission by week sixth and maintained the remission for the next 90 weeks. At the time of last evaluation, tests, including C-reactive protein and fecal calprotectin, were within normal limits, complete colonoscopy revealed erythema, edema, mucosal friability, loss of vascular patterns, and pseudo-polyps. The Pediatric Ulcerative Colitis Activity Index and Mayo scores were 5 and 2 points, respectively. No adverse events were documented.

**Conclusion:**

Golimumab has shown potential efficacy and safety in the treatment of ulcerative colitis in children which may indicate a significant future role for subcutaneous golimumab in pediatrics ulcerative colitis.

## 1 Introduction

Ulcerative colitis (UC) is a chronic inflammatory disease affecting the colonic mucosa.^[1]^ In pediatric patients the disease is more extensive and linked with severe exacerbations,^[2]^ and treatment with corticosteroids, 5-aminosalicylic acid , and thiopurines,^[3]^ or even tumor necrosis factor antagonists (anti-TNF) may fail. Both the European Medicines Agency and the American Food and Drug Administration have authorized infliximab and adalimumab as anti-TNF agents for the treatment of pediatric UC.^[4,5]^ Management of UC flares is based on hospitalization, intravenous fluids with correction of an electrolyte imbalance, excluding mimetics diseases such as Cytomegalovirus and *Clostridium difficile*. If intravenous corticosteroids fail to induce response after three days of treatment, we resort to either adding or escalation anti-TNF medication, and cyclosporine or tacrolimus can also be used for treatment. Expect for infliximab and steroids, the rest of the treatments were unavailable in Syria,^[4]^ and the surgery is considered the last therapeutic option in the treatment of UC flares.^[6]^ Golimumab is a new fully human, monoclonal anti-TNF indicated for the treatment of adults with rheumatoid arthritis,^[7–11]^ psoriatic arthritis,^[12,13]^ ankylosing spondylitis,^[14,15]^ and ulcerative colitis.^[16,17]^ In children, golimumab had been used exclusively for rheumatologic diseases.^[18]^

We used an adult induction/loading dose of subcutaneous (SC) golimumab (200 mg at week 0, followed by 100 mg at week 2, then 50 mg every 4 weeks) to induce and maintain remission in a steroid-refractory, low-weight, biologically experienced child with severe ulcerative colitis flare for >90 weeks.

## 2 Case presentation

**Figure 1:**
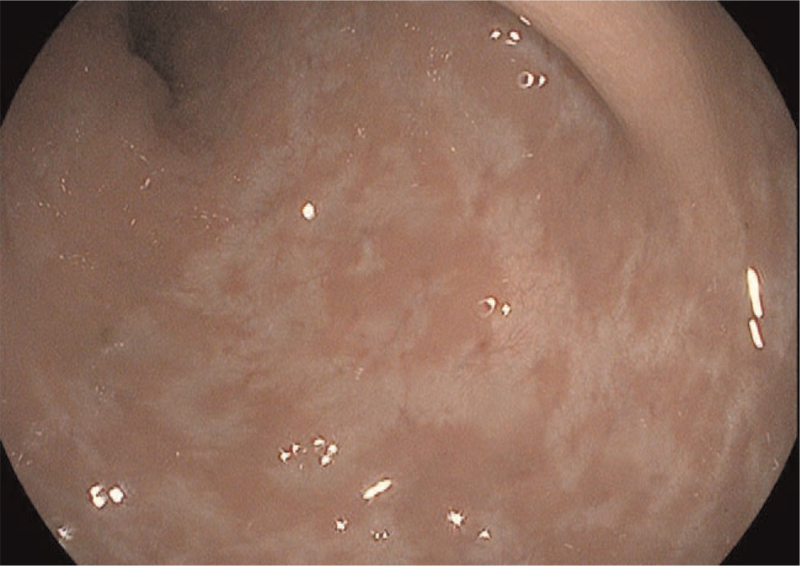
Erythema, edema, mucosal friability and loss of vascular pattern.

**Figure 2:**
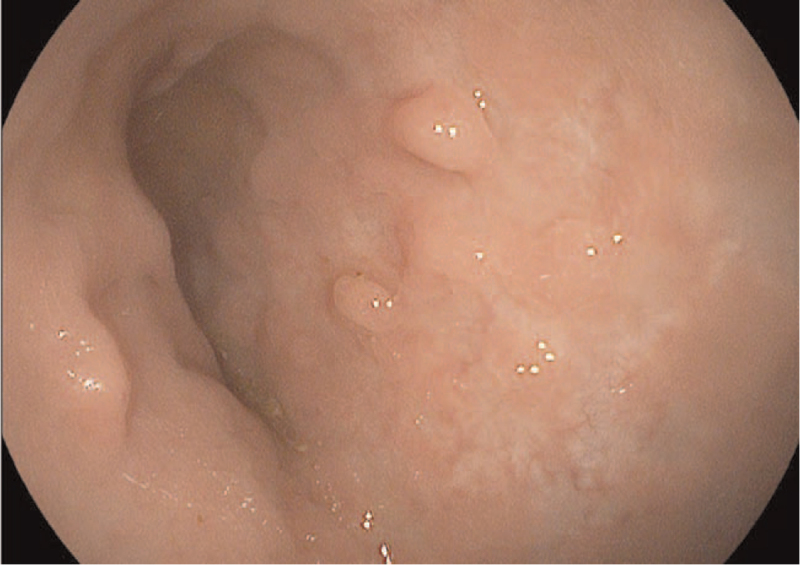
Pseudo polyps.

We had hospitalized of 13-year-old Syrian boy due to pancolitis flare, with no surgical history. The UC diagnosis was established a year ago. His past medications included mesalamine 80 mg/kg/day,^[5]^ azathioprine up to 2.5 mg/kg/day,^[5,19]^ and prednisone 1 mg/kg once daily up to 40 mg during flares then tapering. He began infliximab with an induction dose of 5 mg/kg at 0, 2, and 6 weeks followed by 5 mg/kg every eight weeks. The patient could not maintain a steroid-free clinical remission; thus infliximab was escalated into 5 mg/kg every 6 weeks at week 24, then 5 mg/kg every 4 weeks at week 30.^[20]^ During the course of 40 weeks, he received 8 doses of infliximab. He presented with tachycardia, abdominal tenderness, and 6 to 7 times nocturnal bloody diarrhea. The estimated pediatric ulcerative colitis activity index (PUCAI) was 80 points indicating severe disease,^[21]^ whereas the Mayo score was 11 points. (Sigmoidoscopy revealed ulceration and spontaneous bleeding).^[22]^ We confirmed oral medications compliance by counting both consumed and leftover pills. His physical examination was normal, his body mass index was 17.3 kg/m^2^, and his body surface area was 1.43 m^2^ (Mosteller formula)^[23]^ (weight = 42 kg, height = 156 cm). Initial tests showed anemia, hypoalbuminemia, leukocytosis, an elevation in C-reactive protein (CRP) and fecal calprotectin. Stool studies including routine stool cultures, stool *Clostridium difficile* toxin, testing for *Escherichia coli* O157:H7, and cryptosporidium, microscopy for ova and parasites were all came back negative. Sigmoidoscopy revealed multiple large ulcerations and spontaneous bleeding, and colon biopsies were negative for Cytomegalovirus and *Clostridium difficile* infection. The child did not recover after 5 days of hydrocortisone (300 mg/day in divided doses every 8 hours). Infliximab escalation failed to maintain remission, and cyclosporine, tacrolimus, and adalimumab were all unavailable in Syria. Child's Parents opted against colectomy as a therapeutic option. Although golimumab is not indicated in pediatric UC,^[24]^ we used 200 mg of SC golimumab in week 0, then 100 mg in week 2 followed by 50 mg every 4 weeks until now. The child discharged a week after the loading dose with mild abdominal pain, partially formed stool with limited diarrhea and decreased rectal bleeding (PUCAI = 45 points, moderate disease). Golimumab succeed to treat severe UC flare on biological experienced child. We maintained mesalamine and azathioprine and began tapering prednisone, he returned after two weeks from the first dose for the second induction dose and reassessment. PUCAI was 35 points indicating moderate disease. Clinical response to golimumab is assessed at week 6,^[16,25]^ which is defined by 20 points decrease in PCDAI score.^[26]^ (the child did not complain of abdominal pain or nocturnal stool, he had two times diarrhea partially formed stool and a small amount of rectal bleeding. His partial Mayo score and PUCAI were 6 and 20 points, respectively). Azathioprine was discontinued one year after starting golimumab. The patient sustains remission as we evaluate him every 4 weeks with clinical index (PUCAI, partial Mayo score) and fecal calprotectin every 3 to 6 months.^[27]^ After 90 weeks, due to the COVID-19 epidemic in Syria during 2020,^[28]^ the child had undergone a complete colonoscopy in addition to clinical and laboratory evaluation. The child had no complaints about one to two formed stools; abdominal ultrasound was normal, laboratory studies including complete blood count, CRP, and fecal calprotectin were within normal limits. Complete colonoscopy revealed erythema, edema, loss of vascular pattern (Fig. 1), and pseudo-polyps (Fig. 2). Table 1 shows the difference in patient's tests before starting golimumab versus week 90 after starting golimumab. The child PUCAI and Mayo scores were 5 and 2 point, respectively, consistent with clinical remission which is defined by either a Mayo score of ≤2 points, with no individual sub-score >1,^[29,30]^ or PUCAI <10 points.^[4]^ Until now, the child continues to take SC golimumab according to the regular schedule at a dose of 50 mg every 4 weeks, with no side effects.

**Table 1:** Comparison of test results before and after golimumab treatment.

Test	Before golimumab	After 90 wk of golimumab treatment	Units	Normal value
WBC	18900	7200	mm^3^	4500–10,500
CRP	72	4	mg/Ll	0–5
Fecal calprotectin	625	10	mg/kg	Up to 120
Red blood cells	3.60 × 10^6^	3.90 × 10^6^	mm^3^	(3.7–4.9) × 10^6^
Hemoglobin	8.7	12.90	g/dL	11–14.3
MCHC	33.46	32.25	%	32%–36%
MCV	72.22	81.63	Fl	80–94
MCH	24.17	26.33	Pg	27–31
Platelets	503 × 10^3^	130 × 10^3^	mm^3^	(150–450) × 10^3^
ALT/SGPT	5	16	U/L	5–40
AST/SGOT	7	10	U/L	5–40
Gamma G.T	12	17	mg/dL	8–61
Alkaline Phosphatase	64	67	U/L	40–129
Calcium	7.1	8.9	mg/dL	8–10.4
Ca++	0.9	1.1 (4.69mg/dl)	mmol/L	0.85–1.15
Phosphorous	3.3	3.2	mmol/L	2.7–5
Sodium	138	135	mmol/L	134–146
Potassium	2.5	3.8	mmol/L	3.5–5.0
Iron (Fe)	25	70	μg/dL	40–148
UIBC	86	302	μg/dL	125–345
LDH	236	125	U/L	100–225

ALT = alanine aminotransferase, AST = aspartate aminotransferase, CRP = C-reactive protein, LDH = lactate dehydrogenase, MCH = mean corpuscular hemoglobin, MCHC = mean corpuscular hemoglobin concentration, MCV = mean corpuscular volume, SGOT = serum glutamate oxaloacetate transaminase, SGPT = serum glutamate pyruvate transaminase.

## 3 Discussion

UC is a diffuse mucosal colon-limited inflammation. The typical presenting symptoms are diarrhea, rectal bleeding, and abdominal pain. Treatment aims to achieve clinical response and clinical remission. In a step-up approach that is commonly used in developed countries,^[31]^ chronically active UC treated with the 5-aminosalicylic acid and immunomodulators for instance azathioprine or 6-mercaptopurine, and in case of failure to maintain remission then thiopurines metabolites are measured,^[32]^ and in case of compliance, anti-TNF is added.^[4,5]^ TNF is a cytokine that center on many aspects of an inflammatory response^[33]^; thus, anti-TNF aids in the treatment of many mediated diseases.^[34]^ Anti-TNF treatment carries with it many risks including an increased risk of incidence of opportunistic infection like tuberculosis, neurological diseases like multiple sclerosis, cardiac diseases like congestive heart failure, hematological disease, malignancy, and autoimmune disease.^[35]^ Infliximab is anti-TNF indicated to treat inflammatory bowel diseases in both adults and children. Infliximab response to treatment is evaluated in the eighth week, and failure to induce clinical response results in primary nonresponse, while loss of efficacy over time is a loss of response.^[36]^ In both cases, Infliximab and infliximab antibodies levels should be measured, and in case of undetectable infliximab titers in the presence of positive infliximab antibodies then infliximab escalation is needed, and if escalation fails, we switch to alternative biological.^[4]^ Measuring infliximab and infliximab antibodies titers had several drawbacks including the use of different techniques for detecting antibodies, reported results are in nonstandard form, there is no consensus about best accurate and clinically beneficial method.^[37]^ Furthermore, testing levels of infliximab and infliximab antibodies were unavailable in many countries. In addition to all of the above a systematic review conducted by Freeman et al showed that both anti-TNF and anti-TNF antibody levels had uncertain predictive accuracy to expect a loss of response^[38]^; alternatively, high CRP levels are associated with low infliximab serum levels and high infliximab antibodies concentrations. Although high CRP levels alone didn’t confirm the loss of response or antibodies existence.^[39]^ SC golimumab is a human anti-TNFα IgG1κ monoclonal antibody indicated for the treatment of many TNF mediated diseases. It is available in 100 mg or 50 mg prefilled auto-injector syringe.^[40]^ Golimumab is safe and effective in children with active polyarticular juvenile idiopathic arthritis with a weight-based dosage (30 mg/m^2^ of the body surface area; maximum 50 mg/dose). The evidence came from a large multi-center, double-blind, randomized trial conducted by Brunner et al. Authors conclude that naïve children with active polyarticular-course juvenile idiopathic arthritis may benefit from a weight-based dosage of SC golimumab.^[18]^ Although the efficacy and safety of golimumab in pediatric ulcerative colitis patients have not been established, the only evidence came from the Hyams et al's study. They conducted an open-label study to assess the pharmacokinetics (week 0–14) of weight-based dosage of subcutaneous golimumab (<45 kg [90/45 mg/m^2^]; ≥ 45 kg [200/100 mg]) to treat moderate to severe ulcerative colitis biologically naïve children. They found that the pharmacokinetics of adults and pediatrics were similar, and a weight-based dosage of golimumab showed clinical benefit promise in biologically naive pediatric with moderately-to-severely active ulcerative colitis.^[24]^ Comparable to Hyams et al, in this case the child was a secondary nonresponder to infliximab (biological-experienced) with a steroid-refractory flare. We used 200/100 mg as a loading/induction dosage since we were incapable to assess golimumab or golimumab antibodies concentration, and serum golimumab concentrations were higher in a patient who had a high induction dose.^[24,41,42]^ The child was followed up on golimumab with a dose every 4 weeks for >90 weeks. The washout period aims to overcome the overlap between biological therapies, wasn’t required in the case of losing efficacy.^[43,44]^ Golimumab was well tolerated and resulted in quick and significant improvement as well as important endoscopic improvement, presence of pseuopolyps associated with mucosal healing.^[45]^ Concomitant azathioprine with golimumab reduced the immunogenicity but increased the risk of death of lymphoma in young patients.^[17,46–48]^ We did not record any adverse events even though golimumab was maintained for >90 weeks during the COVID-19 pandemic.^[49,50]^

## Acknowledgments

On behalf of all the contributors, the author will act as guarantor and will correspond with the journal from this point onward.

## Author contributions

**Conceptualization:** Marouf Mouhammad Alhalabi.

**Writing – original draft:** Marouf Mouhammad Alhalabi.

**Writing – review & editing:** Marouf Mouhammad Alhalabi, Kamal Othman Alaa Eddin, Khaled Mouhammad Cheha, Ahmad Jamil Abbas.
